# Retraction Note to: Coffee component hydroxyl hydroquinone (HHQ) as a putative ligand for PPAR gamma and implications in breast cancer

**DOI:** 10.1186/s12864-022-08371-5

**Published:** 2022-02-14

**Authors:** Babita Shashni, Karun Sharma, Rumani Singh, Kishore R. Sakharkar, Sarinder K. Dhillon, Yukio Nagasaki, Meena K. Sakharkar

**Affiliations:** 1 Graduate School of Life and Environmental Sciences, University of Tsukuba, Tsukuba, Ibaraki Japan; 2 AIST, Tsukuba, Japan; 3 OmicsVista, Singapore, Singapore; 4 Institute of Biological Sciences, Faculty of Science, University Malaya, Kuala Lumpur, Malaysia; 5 Department of Materials Sciences, Graduate School of Pure and Applied Sciences, University of Tsukuba, Ibaraki, Japan; 6 Master’s School of Medical Sciences, Graduate School of Comprehensive Human Sciences, University of Tsukuba, Ibaraki, Japan; 7 Satellite Laboratory, International Center for Materials Nanoarchitectonics (WPI-MANA), National Institute for Materials Science (NIMS), University of Tsukuba, Ibaraki, Japan; 8 Department of Biotechnology, University of Pune, Pune, India; 9 Drug Discovery and Development Research Group, College of Pharmacy and Nutrition, University of Saskatchewan, Saskatoon, Canada

**Retraction Note to: BMC Genomics 14, S6 (2013)**

**https://doi.org/10.1186/1471-2164-14-S5-S6**

The Editor is retracting this article because of significant overlap of text with [1] and [2]. Babita Shashni agrees with this retraction. Yukio Nagasaki and Meena K Sakharkar do not agree with this retraction. Karun Sharma, Kishore R Sakharkar and Sarinder K Dhillon have not responded to correspondence from the Editor about this retraction. The Editor was not able to obtain a current email address for Rumani Singh.

## References

[1] Pelicano H, Carney D, Huanga P. ROS stress in cancer cells and therapeutic implications. Drug Resistance Updates. 2004;7(2):97-110. 10.1016/j.drup.2004.01.004 15158766

[2] Shashni B, Sakharkar KR, Nagasaki Y, Sakharkar MK. Glycolytic enzymes PGK1 and PKM2 as novel transcriptional targets of PPARγ in breast cancer pathophysiology. Journal of Drug Targeting. 2013;21(2):161-74. 10.3109/1061186X.2012.736998 23130662

